# Rickettsiae in Gulf Coast Ticks*,* Arkansas, USA

**DOI:** 10.3201/eid1605.091314

**Published:** 2010-05

**Authors:** Rebecca Trout, C. Dayton Steelman, Allen L. Szalanski, Phillip C. Williamson

**Affiliations:** University of Arkansas, Fayetteville, Arkansas, USA (R. Trout, C.D. Steelman, A.L. Szalanski); University of North Texas Health Science Center, Fort Worth, Texas, USA (P.C. Williamson)

**Keywords:** Rickettsiae, vector-borne infections, Rocky Mountain spotted fever, Amblyomma maculatum, Rickettsia parkeri, Rickettsia amblyommii, Gulf Coast, ticks, Arkansas, dispatch

## Abstract

To determine the cause of spotted fever cases in the southern United States, we screened Gulf Coast ticks (*Amblyomma maculatum*) collected in Arkansas for rickettsiae. Of the screened ticks, 30% had PCR amplicons consistent with *Rickettsia parkeri* or *Candidatus* Rickettsia amblyommii.

The Centers for Disease Control and Prevention identified Arkansas as a leading state for the incidence of Rocky Mountain spotted fever (causative agent *Rickettsia rickettsii*) and reported >15 cases per 1,000,000 persons in 2002 (*1*). Given the known cross-reactivity of serologic testing results for spotted fever group (SFG) rickettsia, it is unclear if cases outside the natural range of the vectors for *R. rickettsii* are misdiagnosed, if the pathogen is less virulent than previously suggested, or if additional rickettsiae are responsible for pathogenesis (*2*).

Recently, the Gulf Coast tick (*Amblyomma maculatum*) was identified as the primary vector of *R. parkeri*, a newly described pathogen that causes disease symptoms similar to Rocky Mountain spotted fever (*3*). *R. parkeri* has previously been identified in *A. maculatum* tick specimens collected in the southeastern United States (*4*) and from a human biopsy specimen in Virginia, USA (*5*). We have identified *A. maculatum* ticks collected from canids, felids, white-tailed deer, and a cow from locations throughout Arkansas (*6*). Notably, *R. amblyommii* has been identified as a potential pathogen and is found in lone star ticks (*A. americanum*) (*7*,*8*). We report the presence of DNA consistent with that of *Candidatus* Rickettsia amblyommii and *R. parkeri* in *A. maculatum* ticks in Arkansas.

## The Study

We screened 112 *A. maculatum* ticks collected during March 2006–January 2008 from 22 dogs (*Canis lupus familiaris*) and 95 *A. maculatum* ticks collected during the 2008 hunting season from 52 white-tailed deer (*Odocoileus virginianus* Boddaert) for rickettsial DNA. Collectors removed specimens; stored them in vials containing 100% ethanol; and recorded tick collection date, location, and host (*6*). Ticks were identified by species, sex, life stage, and engorgement (*9*). Each sample was bisected longitudinally with a razor blade and subjected to the extraction procedure by using QIAGEN DNeasy (QIAGEN, Valencia, CA, USA) following the manufacturer’s protocols.

Tick DNA extracts were screened for SFG *Rickettsia* spp. DNA by PCR by using genus-specific primers for the citrate synthase (*gltA)* (*10*) and rickettsial outer membrane protein B (*rompB*) (*11*) genes. Reaction products were analyzed (*12*), and positive amplicons for *gltA* (513 bp) and *rompB* (578 bp) were sent to the University of North Texas Health Science Center (Fort Worth, TX, USA) for sequence determination. At least 1 amplicon from each host was sequenced to determine the *Rickettsia* species identity. PCR products were hydrolyzed with ExoSAP-IT (USB Corporation, Cleveland, OH, USA), and sequence determination was performed by using a BigDYE Terminator v.3.1 Cycle Sequencing Kit (Applied Biosystems, Inc., Foster City, CA, USA) followed by capillary electrophoresis on an ABI PRISM 310 Genetic Analyzer (Applied Biosystems, Inc.) (*13*).

Sequences were edited, aligned, and analyzed with Sequencher 4.7 (Gene Codes Corporation, Ann Arbor, MI, USA) and compared with sequences in GenBank (National Center for Biotechnology Information, Bethesda, MD, USA). BEAST version 1.4.2 software (http://beast.bio.ed.ac.uk/Main_Page) was used to infer phylogenetic relationships and create dendrograms (*14*). The consensus tree ran for 10^6^ generations with a burn-in of 2 × 10^4^. Established methods were used (*12*) to conduct parsimony bootstrap and maximum-likelihood analyses. Maximum-likelihood and unweighted parsimony analyses on the alignments were performed by using the branch and bound algorithm of PAUP* 4.0b10 (http://*paup.csit.fsu.edu).* Outgroup taxa were obtained from GenBank.

Of the 207 ticks, 62 were positive for *Rickettsia* spp. DNA by PCR. Nineteen ticks were positive for *gltA* only, 12 were positive for *rompB* only, and 31 were positive for both genes (Table). Of the ticks collected from white-tailed deer, 28 were positive, and those amplicons were 100% homologous with *Candidatus* Rickettsia amblyommii from GenBank (FJ455415, EU7228827, AY388899) (Table, Figure). Of the positive ticks collected from dogs, 3 had sequences with 100% similarity to either *rompB* (AF123717) or *gltA* (EF102236) of *R. parkeri*. A single tick (unengorged male) had a sequence 98% similar to GenBank sequences EF219464 (*rompB*) and EF451001 (*gltA*). The remaining 30 ticks collected from dogs that were positive all produced amplicons with 100% sequence identity to *Candidatus* R. amblyommii *gltA* (EF450708). However, *rompB* sequences generated from the same sample set demonstrated greater diversity (Table, Figure).

**Table:** Gulf Coast ticks (*Amblyomma maculatum*) collected from white-tailed deer and dogs, Arkansas, USA, 2006–2008*

Source	No. tested	No. (%) positive	Blood meal	*gltA* positive	*rompB-*positive groups					*gltA-* and *rompB-*positive groups					
					2	3	4	5		1	2	3	4	6	NS
White-tailed deer															
Nymph	8	0	Yes												
			No												
Male	46	15 (33)	Yes								2				2
			No	5	2						4				
Female	41	13 (32)	Yes	2	2										
			No	3	3						3				
Total collected from deer	95	28 (29)		10	7	0	0	0		0	9	0	0	0	2
Dogs															
Nymph	8	2 (25)	Yes												
			No	1							1				
Male	95	28 (29)	Yes								1				1
			No	8	1	1	1	1			8	2	3	1	
Female	9	4 (44)	Yes												
			No		1					3					
Total collected from dogs	112	34 (30)		9	2	1	1	1		3	10	2	3	1	1
Total ticks collected	207	62 (30)		19	9	1	1	1		3	19	2	3	1	3

*Of 207 ticks collected, 30% were positive for rickettsiae. Ticks were positive for citrate synthase (*gltA)* gene only, rickettsial outer membrane protein B (*rompB)* gene only, or both genes; *rompB* sequences fell within 6 sequence groups. Ticks were collected from canids during March 2006–January 2008 and from deer during the 2008 deer hunting season. NS, not sequenced.

In total, 3 ticks collected from 3 different canine hosts produced sequences 100% identical to those of *R. parkeri rompB* (AF123717) and *gltA* (EF102236). *Candidatus* R. amblyommii sequences were identified in 29 ticks collected from 13 dogs and 25 ticks collected from 25 deer. The resulting Bayesian tree showed weak support (consistency index 0.792, tree length 159) (Figure). Neighbor-joining and maximum-likelihood trees supported the GenBank homologies.

**Figure:**
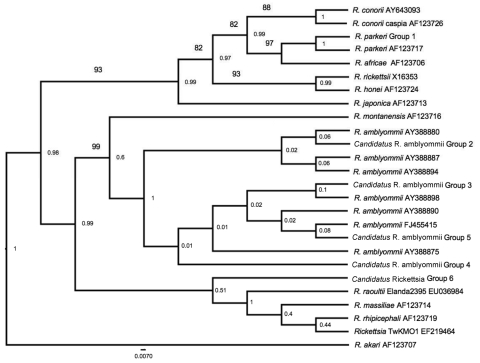
Phylogenetic relationship of 6 rickettsial outer membrane protein B rickettsiae groups (578 bp) identified in *Amblyomma maculatum* ticks collected in Arkansas and similar rickettsiae identified from GenBank. The tree was constructed by using the maximum-likelihood and maximum-parsimony analysis in BEAST 9 (http://beast.bio.ed.ac.uk/Main_Page) Numbers on lines are bootstrap support values >75 and numbers at nodes are posterior values. Scale bar indicates nucleotide substitutions per site.

## Conclusions

We report the identification of SFG rickettsiae in *A. maculatum* ticks collected from Arkansas, specifically *R. parkeri, Candidatus* R. amblyommii, and an uncharacterized *Rickettsia* sp. sequence with high homology to GenBank sequence no. EF219464. Identification of these rickettsiae may be a public health concern given their recent association with cases of spotted fever (*4*,*7*,*8*). The risk for spotted fever transmission to humans is unknown but may be of concern to public health officials in Arkansas because of canid–human relationships and habitat fragmentation that has moved deer ranges closer to human habitation. Additional investigations of the distribution of *A. maculatum* ticks, the pathogenesis of Rocky Mountain spotted fever, and the ticks’ relationship to human disease should be conducted.
